# Single molecule localization microscopy coupled with touch preparation for the quantification of trastuzumab-bound HER2

**DOI:** 10.1038/s41598-018-33225-0

**Published:** 2018-10-11

**Authors:** Steven J. Tobin, Devin L. Wakefield, Veronica Jones, Xueli Liu, Daniel Schmolze, Tijana Jovanović-Talisman

**Affiliations:** 1 Department of Molecular Medicine, Beckman Research Institute, City of Hope Comprehensive Cancer Center, Duarte, CA 91010 USA; 2 Department of Surgery, City of Hope Comprehensive Cancer Center, Duarte, CA 91010 USA; 3 Division of Biostatistics, City of Hope Comprehensive Cancer Center, Duarte, CA 91010 USA; 4 Department of Pathology, City of Hope Comprehensive Cancer Center, Duarte, CA 91010 USA

## Abstract

All breast cancers are assessed for levels of human epidermal growth factor receptor 2 (HER2). Fluorescence *in situ* hybridization (FISH) and immunohistochemistry are currently used to determine if a patient is eligible for anti-HER2 therapy. Limitations of both tests include variability and relatively long processing times. Additionally, neither test determines whether HER2 contains the extracellular domain. While truncated in some tumors, this domain is required for binding of the therapeutic antibody trastuzumab. Here, trastuzumab was used to directly detect HER2 with quantitative single molecule localization microscopy (qSMLM). In proof of concept studies, our new method rapidly quantified both HER2 density and features of nano-organization. In cultured cells, the method was sensitive to subtle variations in HER2 expression. To assess patient samples, we combined qSMLM with tissue touch preparation (touch prep-qSMLM) and examined large areas of intact membranes. For cell lines and patient samples, HER2 copy numbers from FISH showed a significant positive correlation with detected densities from qSMLM and trended with HER2 cluster occupancy.

## Introduction

In the United States, nearly one in eight women will develop invasive breast cancer, which is the second leading cause of female cancer-related deaths^1^. Approximately 25% of breast cancers show elevated expression of human epidermal growth factor receptor 2 (HER2)^2^ and are denoted *HER2-positive* cancers. This receptor is an important prognostic and predictive biomarker^3,4^. Before the advent of HER2 directed therapies, patients with HER2-positive breast cancer faced a relatively poor prognosis^2,5^. Today, HER2-positive tumors can be treated with anti-HER2 agents. The clinical monoclonal antibody trastuzumab (Herceptin) is one important example. Trastuzumab alone^6^ or in combination with other therapeutics^7,8^ can significantly improve patient outcomes^9^.

To determine the appropriate therapy for breast cancer patients, the HER2 status of their tumor must be accurately established. Current laboratory practice follows joint 2018 guidelines from the American Society of Clinical Oncologists (ASCO) and the College of American Pathologists (CAP)^10^. These guidelines endorse two methods for HER2 testing: fluorescence *in situ* hybridization (FISH), which assesses HER2 gene amplification, and immunohistochemistry (IHC), which assesses HER2 protein expression. By pairing IHC and FISH, individual shortcomings in these tests are minimized and the result is a more accurate assessment of HER2 status^11^.

While a 95% concordance between the two methods is recommended, such agreement is often not achieved^12,13^. Both pre-analytical and analytical factors contribute to testing variability. For example, IHC results depend on both formalin fixation time^14^ and the choice of anti-HER2 antibody^15,16^. Moreover, the results are semi-quantitative and rely on pathologist assessment, which is subjective^17^. Consequently, approximately 20% of IHC results are considered inconclusive (equivocal)^10,18^. Discrepancies between IHC and FISH may also reflect tumor biology. For example, in a small percentage of breast cancers, activating HER2 mutations exist without gene amplification^19^. In these cases, IHC results are positive whereas FISH results are negative. Such scenarios underscore the need for new ways to quantify HER2 protein levels.

Routine analysis of breast cancer biopsies and surgical resections generally focus on protein expression at the cellular level. However, proteins execute their functions at the molecular level. Features in the molecular (nanoscale) distribution of HER2 may contain clinically useful information. A number of studies have used single molecule localization microscopy (SMLM) techniques^20–25^, such as direct stochastic optical reconstruction microscopy (dSTORM)^23^, to evaluate the distribution of proteins in cultured cells^26–28^. These techniques are also compatible with tissue specimen imaging. SMLM was used to detect HER2 in paraffin embedded tissue sections^29^ and to determine how G-protein coupled receptors organize in fresh-frozen human tumor samples^30^.

Here, we present a new quantitative SMLM (qSMLM) approach to assess HER2 in cultured breast cancer cell lines and freshly excised breast cancer tissues. We quantified HER2 density and determined features of HER2 membrane nano-organization. Fluorescently labeled trastuzumab was used for SMLM detection. This approach reduced sample preparation time and ensured that the detected HER2 contained the trastuzumab binding domain. Minimal effort was needed to prepare patient samples because tissues were acquired by a touch preparation technique. We call this approach “touch prep-qSMLM”. Using this straightforward analytical protocol and algorithms designed for rapid data analysis, quantitative results for patients were obtained within one day. Ultimately, touch prep-qSMLM may clarify ambiguities in HER2 status and help inform therapeutic decisions. Our methodology has the potential to expand the arsenal of technologies^31^ available to physicians for characterizing a wide range of protein and gene signatures in health and disease.

## Results

### Characterization of HER2 density and organization in cells with qSMLM

Breast cancer is a heterogeneous disease comprising multiple subtypes, each with distinct morphologic, genetic, and clinical features^32^. We examined HER2 density in three cultured breast cancer cell lines: BT-474, SK-BR-3, and MDA-MB-468. BT-474 cells are a model for the luminal B breast cancer subtype and SK-BR-3 cells are a model for the HER2-enriched subtype. IHC indicates that the two cell lines are HER2-positive, and FISH yields high and distinct HER2 copy number values^33,34^. MDA-MB-468 cells are classified as the basal-like subtype. IHC indicates these cells as HER2-negative whereas FISH analysis produces a low yet detectable HER2 copy number value^33,34^. We used the distinct HER2 expression levels of these three breast cancer subtypes to examine the sensitivity of our qSMLM approach.

To image these cell lines by SMLM, trastuzumab was labeled with approximately one Alexa Fluor 647 (AF647) dye (see *Methods* for further explanation). The optimal concentration for imaging with trastuzumab-AF647 was determined to be 50 nM. Concentrations below 10 nM did not stain cells as efficiently, whereas concentrations above 50 nM showed significant background signal. As a control, soluble HER2 was premixed with trastuzumab-AF647 prior to cell staining. Under these conditions, negligible AF647 signal was associated with cell lines (Supplementary Fig. 1). These results confirm the specificity of fluorescently labeled trastuzumab for SMLM imaging.

**Figure 1:**
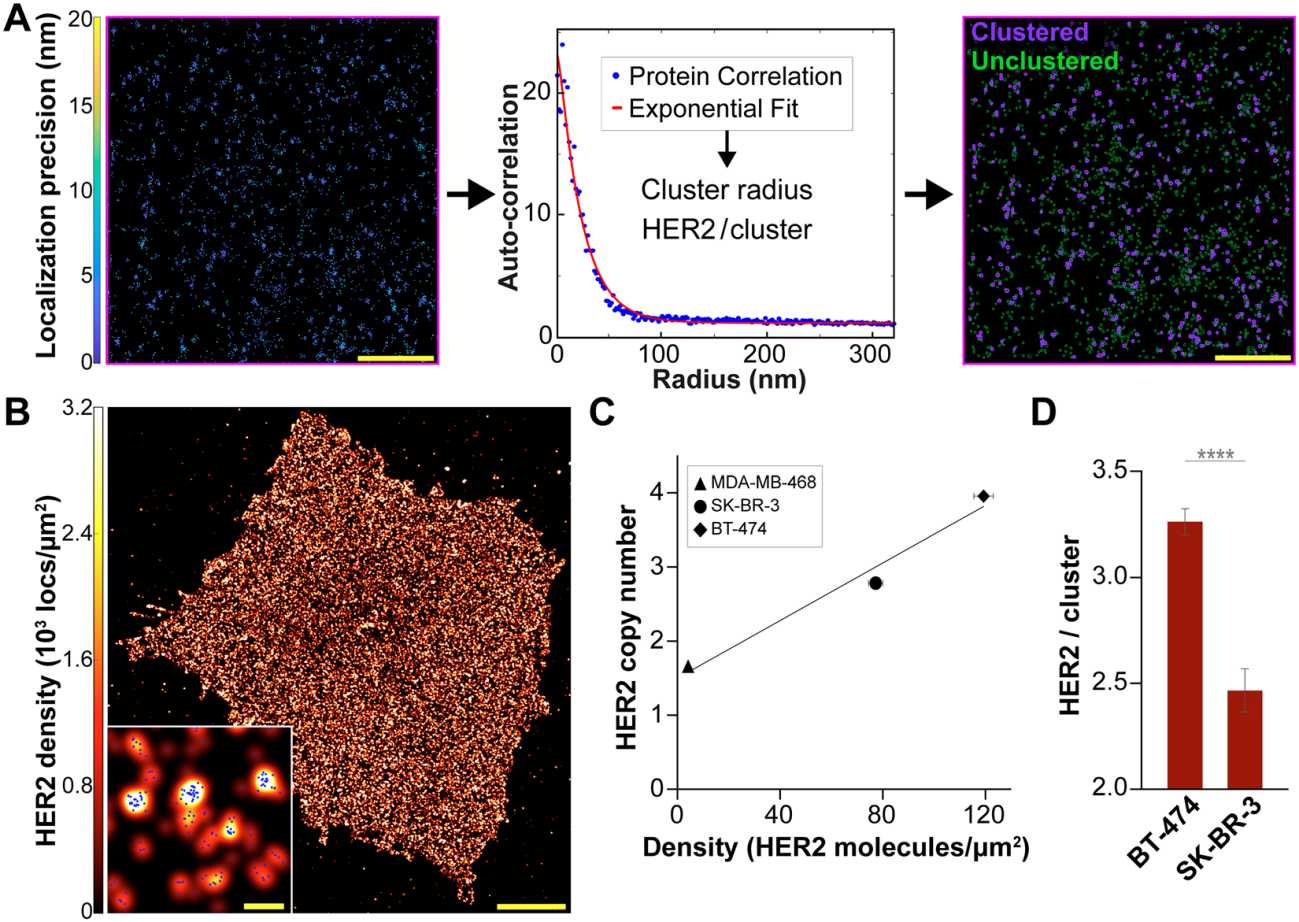
HER2 molecular features in breast cancer cell lines. BT-474, SK-BR-3, and MDA-MB-468 cells were fixed, stained with 50 nM trastuzumab-AF647, and imaged with dSTORM. (A) Outline of qSMLM approach with a representative 20 µm^2^ ROI from a BT-474 cell (scale bar: 1 µm). In the right panel, purple and green circles represent clustered (more than 2 HER2 molecules residing in cluster) and unclustered HER2, respectively. (**B**) BT-474 cell with individual localizations (blue) rendered by a Gaussian blur. The intensity of the Gaussian blur is proportional to the localization density. Scale bar: 5 μm. A zoomed-in region is provided as an inset (scale bar: 100 nm). (**C**) Average detected HER2 density from qSMLM versus HER2 copy number from FISH. Copy number values were taken from the Cancer Cell Line Encyclopedia – Broad Institute^33,34^. The following number of ROIs were used for analysis: MDA-MB-468 (19 cells, 37 ROIs), SK-BR-3 (17 cells, 163 ROIs), and BT-474 (23 cells, 180 ROIs). The best-fit line has R^2^ = 0.96. (**D**) Average number of HER2 receptors per cluster for BT-474 and SK-BR-3 cells calculated by PC analysis. Clustered regions were used for analysis (53 ROIs for BT-474 and 31 ROIs for SK-BR-3); ****p < 0.0001. All error bars represent SEM. Additional statistical considerations are included in Supplementary Table 1.

To account for multiple appearances of a single trastuzumab-AF647 antibody in SMLM images, we experimentally determined the average number of localizations (appearances) of trastuzumab-AF647 using MDA-MB-468 cells (Supplementary Fig. 2). MDA-MB-468 cells were used for this characterization because the surface HER2 expression is very low^35^ and individual receptors are well separated. Next, we determined the density and nano-organization of HER2 in the three cell lines using pair-correlation (PC)^36,37^ analysis. Subsequently, a k-means-like clustering algorithm^38,39^ was used to quantify the fraction of HER2 molecules residing in clusters with more than two receptors (the fraction of clustered HER2). An outline of the overall analysis approach is shown in Fig. 1A using a representative region of interest (ROI) from a BT-474 cell. Briefly, the spatial PC function describes the average probability of finding a protein at a given radial distance away from another protein. The protein auto-correlation function (*g*(*r*)^*protein*^)^36,37^ is given as:

1 $$g{(r)}^{protein}=1+A{e}^{\frac{-r}{\xi }},$$

where *A* is the amplitude, *r* is the search radius in nm, and ξ is the correlation length (cluster radius). When proteins are randomly distributed (no clusters), *A* is equal to zero and the auto-correlation function is approximately a flat line with *g*(*r*)^*protein*^ = 1. When proteins are distributed into random clusters, the protein auto-correlation is greater than 1 at short distances. By applying an exponential decay function fit, quantitative information can be extracted. This includes the size of the clusters and the number of detected proteins per cluster. Our PC analysis and relevant MATLAB code have been previously reported^36,37^. Next, PC results are fed into a k-means-like clustering algorithm to determine the fraction of clustered HER2 (see *Methods* and ref.^39^ for details). MATLAB code is provided in the Supplementary Information as ‘ClusterOccupancy’.

Using qSMLM, we assessed the amount of HER2 in the plasma membrane of the three cultured breast cancer cell lines. To calculate the detected HER2 membrane densities, the total number of localizations were divided by the average number of appearances for a single trastuzumab-AF647. Supplementary Figs 3 and 4 respectively show the distribution of localization precisions and HER2 densities for all cell experiments. Figure 1B and Supplementary Fig. 5A respectively show representative examples of BT-474 cells imaged using 50 and 10 nM trastuzumab-AF647 (numerical findings are shown in Supplementary Table 1). Average detected HER2 densities obtained using 50 nM trastuzumab-AF647 in three cell lines are shown in Supplementary Fig. 6A. The oncogene addicted BT-474 cells had the highest average detected HER2 density of 119 molecules/μm^2^. SK-BR-3 cells had the next highest average detected HER2 density of 77 molecules/μm^2^. Both BT-474 and SK-BR-3 cells had a relatively large variation in detected densities (Supplementary Fig. 4). MDA-MB-468 cells had a low average detected HER2 density of 4 molecules/μm^2^. We plotted the average detected HER2 densities as a function of published values for HER2 copy numbers obtained using FISH^33,34^. Excellent agreement was observed between known values and our results (Fig. 1C).

HER2 clustering on the plasma membrane is influenced by both HER2 overexpression and its ability to associate with other epidermal growth factor receptor (EGFR) family members^40^. Quantitative analysis of SMLM data was used to analyze features of HER2 molecular organization. We examined the number of HER2 receptors per cluster, cluster radius, and the fraction of clustered HER2 molecules. On average, BT-474 cells had more HER2 receptors per cluster than SK-BR-3 cells (3.3 vs 2.5, respectively, Fig. 1D). However, BT-474 cells exhibited both a lower cluster radius (21 nm vs 24 nm for SK-BR-3 cells, Supplementary Fig. 6B) and a lower fraction of clustered HER2 molecules (49% vs 59% for SK-BR-3 cells, Supplementary Fig. 6C). Supplementary Fig. 7 shows the distributions of the HER2 receptors per cluster, cluster radius, and fraction of clustered HER2 for all investigated ROIs. In contrast to the clustering observed for BT-474 and SK-BR-3 cells, no clusters were detected for MDA-MB-468 cells.

**Figure 2:**
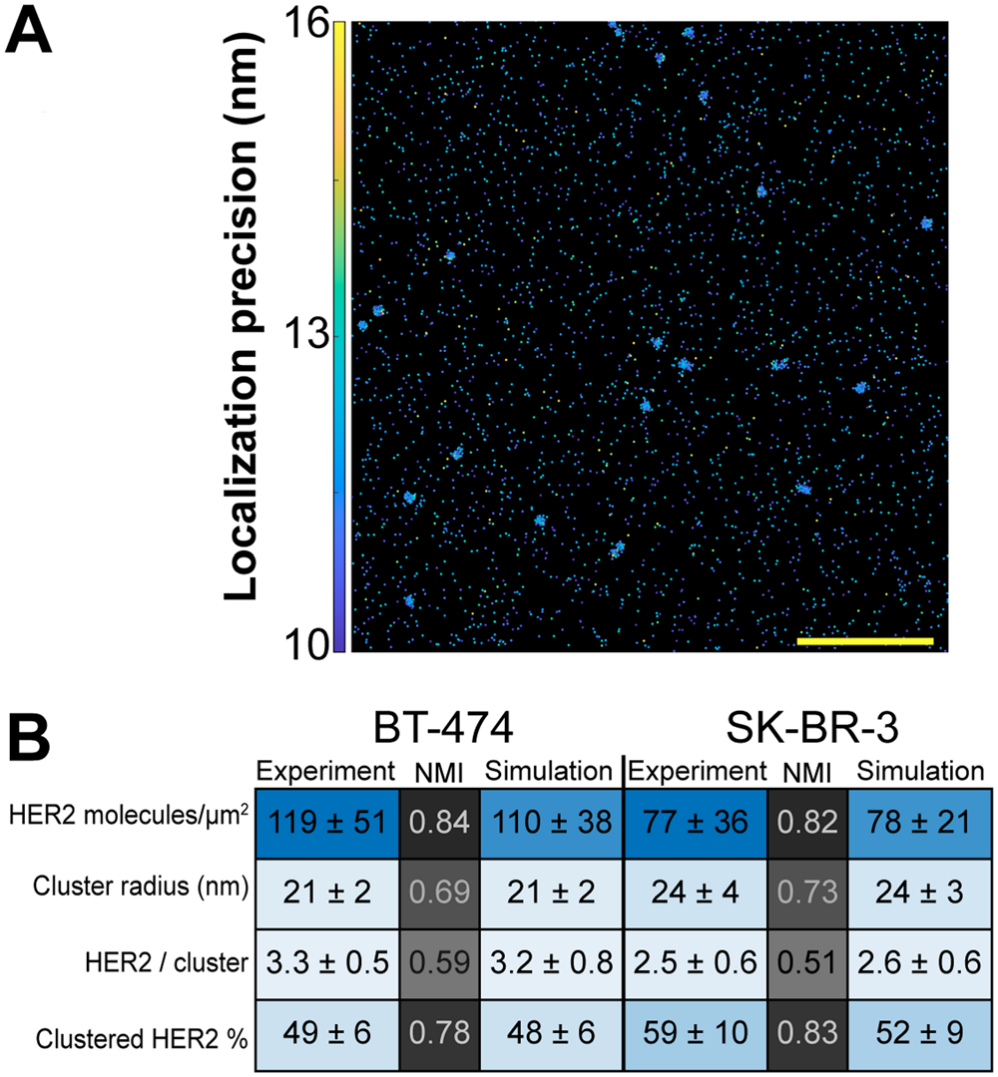
Monte Carlo simulations of SMLM data. Synthetic localization data supports HER2 nano-organization from PC and a k-means-like clustering analysis for BT-474 and SK-BR-3 cell data. (**A**) Simulated ROI shows multiple HER2 clusters. Scale bar: 1 μm. (**B**) Averaged results from BT-474 and SK-BR-3 experimental data (Fig. 1D and Supplementary Fig. 6) and from 100 simulated ROIs for each cell type. Standard deviations are provided and are further used to color in the table (MATLAB *heatmap* function), emphasizing the relative similarity between experimental and simulated data. Normalized mutual information (NMI) scores are provided to compare experimental to simulated data for each cell type. These scores were used to color in the table (MATLAB *heatmap* function). NMI scores range from 0 (no mutual information) to 1 (complete similarity).

Our data on HER2 organization was validated by Monte Carlo simulations (Fig. 2). ROIs with a defined density and number of HER2 clusters were generated to mimic experimentally obtained BT-474 and SK-BR-3 cell data obtained using 50 nM trastuzumab-AF647. Simulation input parameters were established to construct 20 μm^2^ ROIs presenting a range of HER2 cluster radii and cluster compositions (Fig. 2A; see *Methods* for details). Specifically, each synthetic ROI data set incorporated a number of localizations placed randomly within the clusters with some average spatial error. This error was predetermined via the experimental data (~12 nm; Supplementary Table 1). The average number of localizations for a single trastuzumab-AF647 was also used to define the number of localizations residing within a given molecule. 100 ROIs were simulated for each cell type and PC analysis was then performed to extract information on HER2 organization. Figure 2B shows averages from these simulations alongside their experimental counterparts. Additionally, normalized mutual information (NMI) scores were calculated to evaluate the quality of the simulations. NMI scores for BT-474 and SK-BR-3 cells had the following respective values: 0.84 and 0.82 for detected HER2 densities; 0.69 and 0.73 for cluster radius; 0.78 and 0.83 for fraction of clustered HER2; and 0.59 and 0.51 for HER2 receptors per cluster. Since PC analysis results range from approximately two to four for HER2 receptors per cluster (Supplementary Fig. 7A), small deviations between simulated data and experimental data reduced the measure of similarity and produced lower NMI scores for this parameter. Cumulatively, as evidenced through both experimental and simulated data, qSMLM can sensitively detect and effectively evaluate features of HER2 organization.

### HER2 patient tissue preparation and characterization with qSMLM

**Figure 3:**
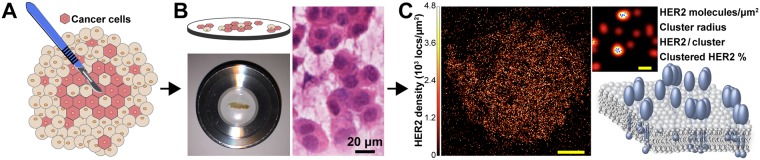
Touch prep-qSMLM experimental design overview. Tumor tissue is carefully cut with a scalpel to detach cells. Tumor tissue on the scalpel blade is brought into contact with poly-L-lysine coated coverslips. qSMLM is performed following a brief incubation to allow tumor tissue to adhere to the coverslip surface, fixation, and staining. (**A**) Illustration of a tumor tissue excision with scalpel. (**B**) Illustration of patient tumor tissue on a coverslip (top left) to be placed inside an imaging chamber (bottom left, Attofluor Cell Chamber) for SMLM. H&E staining of a touch prep from P3 shown by brightfield at 20X magnification (right) collected by the pathology team. (**C**) SMLM image of a HER2-positive cell in patient tissue. Individual localizations were obtained from NIS-Elements (in blue) and rendered by a Gaussian blur. The intensity of the Gaussian blur is proportional to the localization density (full cell, scale bar: 5 μm; zoomed-in region, scale bar: 100 nm). Image analysis is performed to obtain detected receptor density and nano-organization parameters (illustration shown on bottom right). Adobe Illustrator was used to prepare the illustrations shown in **A**, **B** and **C**.

For traditional FISH or IHC testing, a tissue sample is first formalin fixed and paraffin embedded, which may take 12–24 hours^10,18^. Subsequently, a single 4 μm thick tissue slice is used for the assays. To rapidly analyze patient tissues with qSMLM, here we have developed a touch preparation (touch prep) approach, touch prep-qSMLM (Fig. 3). Fresh surgically excised breast specimens were serially sectioned, and areas of tumor were identified by gross examination. A scalpel was used to lightly scrape the tumor and collect tissue (Fig. 3A). Poly-L-lysine coated coverslips were then touched to the tissue accumulated on the edge of the scalpel (Fig. 3B). 2–4 cell monolayers (z-sections) were taken from each tumor. Immediately after collection, the tissue samples were fixed for 30 minutes with a mixture of paraformaldehyde and glutaraldehyde, a standard fixation approach for preparing SMLM samples^37^. Coverslips were incubated with both 50 nM trastuzumab-AF647 and Alexa Fluor 405 (AF405) labeled epithelial marker (GATA3 or Cytokeratin 7) for 1 hour. Following a 10 minute post-fixation step, coverslips were imaged. Altogether, our tissue samples took approximately 3 hours to prepare.

Brightfield imaging was used to confirm tumor cell morphology (Supplementary Fig. 8A). Since tumor mass comprises a heterogeneous mixture of neoplastic epithelial cells and non-epithelial immune and stromal cells^41^, the 405-nm signal from the labeled epithelial markers was used to identify epithelial cells. SMLM was subsequently performed to detect trastuzumab-AF647 bound to these cells. Further details are provided in *Methods*. As with previous touch prep reports^42^, our approach retained crisp cellular details. Intact membranes allowed us to image large areas (Fig. 3C, left). Finally, data was analyzed to obtain detected receptor densities and features of nano-organization (Fig. 3C, right). To validate the specificity of trastuzumab in touch prep-qSMLM, trastuzumab-AF647 was pre-complexed with soluble HER2 and applied to tissue samples. Similar to the blocking controls performed on cell lines, no appreciable signal was detected on these coverslips. An example is shown in Supplementary Fig. 8B. Altogether, touch prep-qSMLM can both rapidly and sensitively detect HER2 in tissues.

### Significant correlation between qSMLM and FISH in patient tissue

**Figure 4:**
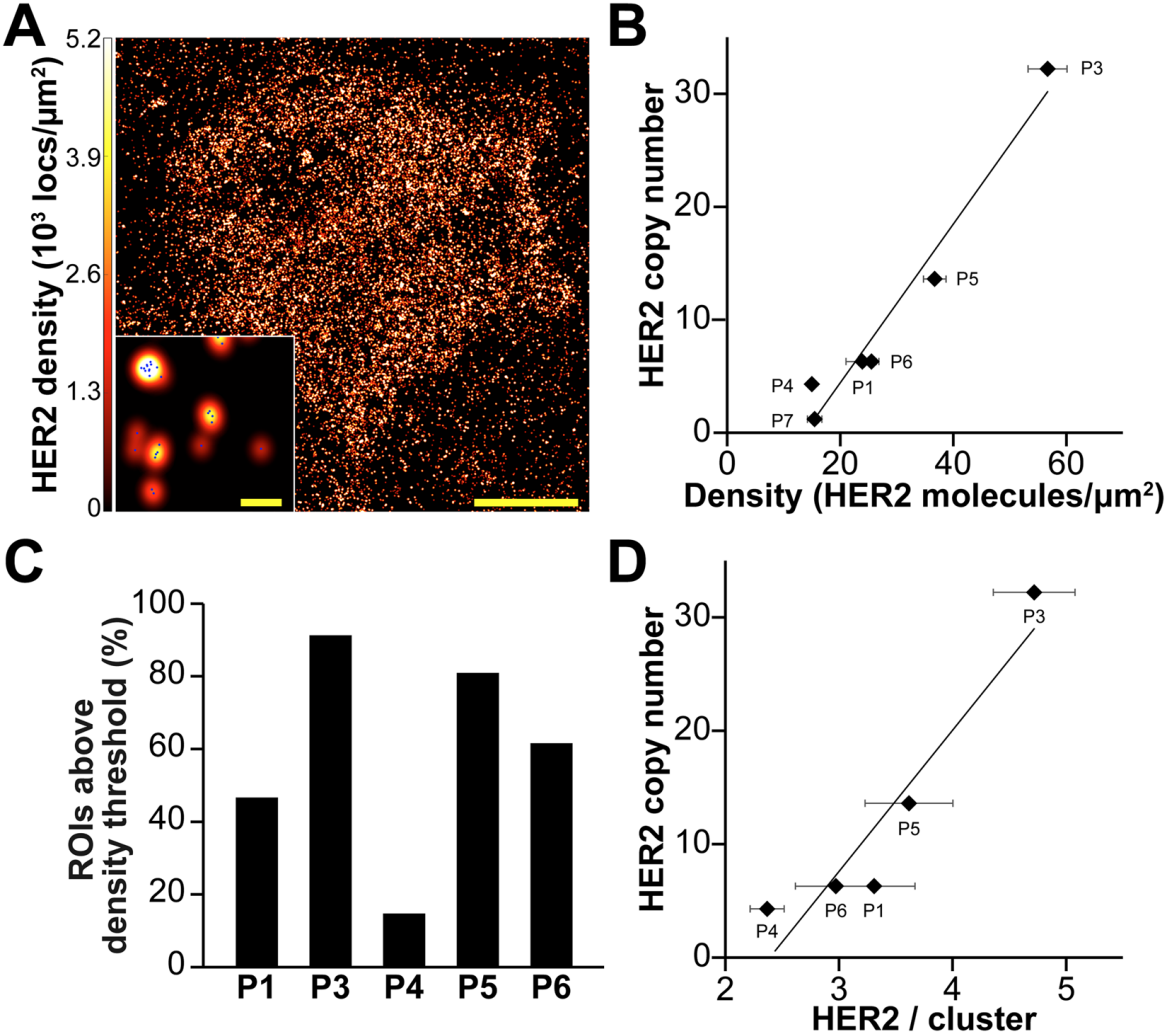
qSMLM results corroborate FISH analysis. (**A**) A touch prep region with individual localizations (blue) rendered by a Gaussian blur. The intensity of the Gaussian blur is proportional to the localization density (full cell, scale bar: 5 μm; zoomed-in region, scale bar: 100 nm). (**B**) Average detected HER2 density from qSMLM versus HER2 copy number from FISH. The following number of ROIs were used for analysis: P1 (30 ROIs), P3 (92 ROIs), P4 (88 ROIs), P5 (131 ROIs), P6 (164 ROIs), P7 (46 ROIs). Complete FISH data was not available for P2. The fitted least-square line has a slope estimate of 0.70, and an intercept estimate of −9.63 (R^2^ = 0.958). (**C**) Fraction of ROIs above HER2 density threshold. (**D**) The average HER2 receptors per cluster versus HER2 copy number from FISH. Clustered regions were used for analysis (19 ROI for P1, 44 ROI for P3, 28 ROI for P4, 10 ROI for P5, and 14 ROI for P6). The fitted least-square line has a slope estimate of 12.45, and an intercept estimate of −29.75 (R^2^ = 0.88). Error bars are SEM. Additional statistical considerations are included in Table 1.

Touch prep-qSMLM was performed on surgical excision specimens from seven breast cancer patients. Six patients were HER2-positive and one patient was HER2-negative based on testing performed on initial core needle biopsy specimens obtained prior to surgery. The imaging team was blinded to all other patient information. For each patient, multiple coverslips and ROIs were imaged. Supplementary Figs 9 and 10 show the distribution of localization precisions and densities for all patient experiments and Table 1 provides a summary of patient characteristics and imaging/analysis statistics. A representative touch prep SMLM image is shown in Fig. 4A. The same procedures described in the cell line studies were used to calculate average detected HER2 densities (Table 1). The obtained values ranged from 15 to 57 molecules/μm^2^. After touch prep-qSMLM, the imaging team was unblinded, and qSMLM detected HER2 densities for the six patients were compared to FISH results. (Complete FISH results were not available for Patient 2). For the five HER2-positive patients, HER2 copy number values ranged from 4.3 to 32.2; for the HER2-negative patient the copy number was 1.2. There was a significant positive correlation between the average detected HER2 densities from touch prep-qSMLM and HER2 copy numbers from FISH (Fig. 4B). The correlation coefficient from the six patients was 0.979 with 95% CI [0.813, 0.998], and the p-value was 0.0007 (Pearson’s correlation test).

**Table 1:** Patient characteristics and data summary.

	P1	P2	P3	P4	P5	P6	P7
Prior Herceptin treatment	no	yes	no	no	no	no	no
Tumor grade	2	2	3	2	2	1	3
Tumor stage	1c	3	1c	2	2	3	2
Lymph node stage	0	0	0	0	N/A	1a	1a
ER (%, intensity)	95%, 2–3+	80%, 3+	60%, 1–2+	>95%, 3+	95%, 3+	90%, 3+	95%, 3+
PR (%, intensity)	>95%, 3+	5%, 3+	0%	75%, 3+	<1%, 1+	0%	75%, 2+
HER2 IHC	3+	3+	3+	3+	3+	3+	0
HER2 FISH copy number	6.3	N/A	32.2	4.3	13.6	6.3	1.2
HER2 FISH ratio	2.4	4.4	4.4	2.5	11.3	1.6	0.7
Molecules/µm^2^ ± SEM	24 ± 3	30 ± 3	57 ± 3	15 ± 1	37 ± 2	26 ± 1	15 ± 1
CV (%)	67	81	59	37	62	42	56
HER2 / cluster ± SEM	3.3 ± 0.4	2.9 ± 0.7	4.7 ± 0.4	2.4 ± 0.1	3.6 ± 0.4	3.0 ± 0.4	monomer
CV (%)	48	60	50	24	34	45	N/A
Cluster radius (nm) ± SEM	25 ± 1	17 ± 1	40 ± 2	18 ± 1	29 ± 2	33 ± 4	N/A
CV (%)	25	13	30	15	21	43	N/A
Clustered HER2 (%) ± SEM	34 ± 5	20 ± 4	67 ± 3	21 ± 2	62 ± 5	59 ± 5	0
CV (%)	61	59	29	54	26	30	N/A
σ (nm) ± SEM	21.1 ± 0.5	14.4 ± 0.2	16.7 ± 0.3	14.8 ± 0.2	13.4 ± 0.1	14.4 ± 0.1	13.3 ± 0.2
CV (%)	12	11	16	12	10	9	11
Coverslips	2	3	3	4	2	4	3
ROIs	30	85	92	88	131	164	46
p-value_split_	0.3	0.2	0.3	0.3	0.5	0.4	0.5

Average values for detected density, HER2 receptors per cluster, cluster radius, fraction of clustered HER2, and localization precision (σ) are provided with SEM and CV. Estrogen receptor (ER) and progesterone receptor (PR) clinical percentage and intensity are reported.

Tumor heterogeneity for HER2-positive patients was assessed by determining the fraction of ROIs with a density above a set threshold. Current clinical guidelines for single-probe HER2 FISH testing define HER2 positivity as an average HER2 copy number of at least six^10,43^. This copy number translates to a density of 22 receptors/µm^2^ for our study based on the correlation in Fig. 4B. The fraction of ROIs with a density above 22 receptors/µm^2^ is shown in Fig. 4C. While P4 had a small fraction of ROIs above the threshold, the other HER2-positive patients had ~50% or more regions above the threshold.

Differences between patients were also observed in HER2 nano-organization. Patient 3 had an average of 4.7 HER2 receptors per cluster, the highest number found in this study. Further, patient 3 had the largest cluster radius (40 nm) and the highest fraction of HER2 receptors in clusters (67%), Table 1. The remaining HER2-positive patients had 2.4 to 3.6 HER2 receptors per cluster, with cluster radii from 17 to 33 nm, and fractions of clustered HER2 encompassing 20% to 62%. Supplementary Fig. 11 shows the distributions for three clustering parameters from the six HER2-positive patients. For clustered ROIs, the average number of HER2 receptors per cluster and HER2 copy number from FISH were correlated (Fig. 4D). The correlation coefficient from the five patients was 0.944 with 95% CI [0.370, 0.996], and the p-value was 0.02 (Pearson’s correlation test). Importantly, for the HER2-negative patient (Patient 7), all regions had a random distribution of HER2. Cumulatively, touch prep-qSMLM can stain for and quantify trastuzumab-bound HER2 in freshly excised tumor tissue. Moreover, our results correlated with FISH analysis and provided insight on HER2 receptor organization.

## Discussion

Fluorescent microscopy methods can now achieve high resolution and have been extensively used to investigate growth factor receptors^44^. Super-resolution microscopy methods have been used to examine HER2 in tissues qualitatively^29,45^. In particular, formalin fixed paraffin-embedded tissue slices were visualized using anti-HER2 antibodies (detecting the intracellular HER2 epitope) and fluorescently labeled secondary antibodies. Rectal cancer tissue sections were imaged with stimulated emission depletion (STED) microscopy. STED images showed HER2 is largely found on the membrane, and that HER2 clusters may be present^45^. Additionally, 2D and 3D SMLM was used to detect HER2 in breast cancer tissues^29^. Fine patterns of HER2 on the membrane were visualized. Both approaches identified blebs containing HER2^29,45^. While these studies clearly demonstrate the utility of super-resolution microscopy methods for the detection of HER2 in tissues, they did not provide details on HER2 membrane density or organization. Other methods, such as localization microscopy and single receptor tracking, have been used to study HER2 expression on the surfaces of breast cancer cells^46,47^. The expression level of this oncoprotein is related to physiological effects and the overexpression of HER2 has been observed in aggressive and invasive breast cancers^2,48^. Moreover, high levels of HER2 can cause the surfaces of breast cancer cells to deform^47^. Such membrane deformations may play a role in the invasive phenotype. These highlighted studies demonstrate the importance of accurately defining the expression of therapeutically relevant oncoproteins.

Here, qSMLM was first used to quantify the amount of HER2 in the plasma membrane of cultured breast cancer cell lines. We generated a nanoscale picture of trastuzumab-bound HER2 (Fig. 1, Supplementary Figs 6, 7). Since trastuzumab-AF647 was used to detect HER2, our method captured only those HER2 variants containing accessible extracellular trastuzumab-binding domain. We examined cell lines with different HER2 expression profiles and observed a relatively large spread in detected HER2 densities for HER2-positive cells (Supplementary Fig. 4). BT-474 cells had the highest average detected HER2 density (119 molecules/μm^2^), SK-BR-3 cells had the next highest density (77 molecules/μm^2^), and low levels were observed in MDA-MB-468 cells (4 molecules/μm^2^), as shown in Supplementary Fig. 6A and Supplementary Table 1. Both BT-474 and SK-BR-3 cells are HER2-positive by IHC^49^. MDA-MB-468 cells have low but detectable copy numbers of the HER2 gene^33,34^, but they are categorized as HER2-negative by IHC^49^. For all three cell lines, previously published HER2 gene copy numbers from FISH^33,34^ correlated with average detected HER2 densities from qSMLM (Fig. 1C). According to these data, our method readily distinguishes subtle differences in oncoprotein expression with high sensitivity.

In addition to accurately determining expression levels, recent high-resolution studies have shed light on HER2 organization on the plasma membrane. Proximity assays and particle tracking have been used to study HER2 dimerization^50–52^. These studies were largely focused on local HER2 organization. However, HER2 overexpression on the surface of cells may cause HER2 to asymmetrically organize into high-density regions^47,53,54^. Such regional differences underscore the importance of understanding the global organization of HER2 across the cell membrane. SMLM methods can provide detailed images of biological structures and information on protein distributions from entire cell membranes^26–28^. Additionally, recent advances^55,56^ have enabled the quantification of protein dimers/oligomers. Thus, SMLM is an excellent tool for molecular assessment of HER2 organization. Here, we combined PC analysis^36,37^ and a k-means-like clustering algorithm^38,39^ to describe features of membrane HER2 clustering. A graphical outline of our analysis approach is shown in Fig. 1A.

When visualized with 50 nM trastuzumab, SK-BR-3 cells had clusters that were 14% larger than BT-474 cells (Supplementary Fig. 6B, Supplementary Table 1). However, these clusters were slightly less dense with fewer HER2 molecules (2.5 vs 3.3 for BT-474 cells, Fig. 1D). In addition, we quantified the fraction of HER2 molecules residing in clusters with more than two receptors. SK-BR-3 cells had more HER2 proteins in clusters than BT-474 cells (59% vs 49%, Supplementary Fig. 6C, Supplementary Table 1). Observed differences in nano-organization between the two cell lines are small but statistically significant. Further studies will be needed to determine the extent to which these differences are physiologically relevant. Accordingly, SK-BR-3 cells have been shown to have more clusters than BT-474 cells^47^. HER2 did not form clusters in MDA-MB-468 cells. Data obtained in breast cancer cell lines were validated using Monte Carlo simulations (Fig. 2). NMI scores for HER2 receptors per cluster were 0.59 and 0.51 for BT-474 and SK-BR-3 cells, respectively. These relatively low values can be attributed to the significant influence of small deviations between data sets (HER2 receptors per cluster results from PC analysis range from only about two to four). All other NMI scores were closer to 1.0 as simulations emulated experimental variation with higher fidelity (from 0.69 to 0.84 for BT-474 cells and from 0.73 to 0.83 for SK-BR-3 cells). Cumulatively, for BT-474 cells and SK-BR-3 cells, clear agreement was observed between experiments and simulations.

HER2 organization on tumor cells may influence oncogenic signaling mechanisms^47^. Even the small differences observed with our method may prove to contain important information on the progression of disease and patient outcomes. Thus, robust methods for quantifying HER2 organization in patient samples may improve clinical diagnoses and facilitate precision medicine. In the breast cancer arena, HER2 diagnostics have been invaluable^31^. They are used for both prognostic and predictive assessments. Moreover, they help guide important decisions for using HER2-targeted therapies. Two HER2 diagnostic tests currently used in the clinic are FISH and IHC. While these tests have had a tremendous impact, four main challenges exist. (1) A concordance of 95% between the two methods is often not achieved^12,13^. (2) Procedures are time intensive, taking 2–3 days for IHC and 7–10 days for FISH. (3) Test results are based on a single 4 µm thick slice of tissue, limiting their ability to fully assess tumor heterogeneity. (4) These tests cannot determine if HER2 contains the accessible trastuzumab binding domain. Since the truncation of extracellular HER2 is associated with trastuzumab resistance^57,58^, it may be valuable to assess the abundance of this domain in the clinic.

Recent attempts to address some aspects of these challenges have used a variety of methods^50,59–65^. Both mass cytometry^61^ and ion beam imaging^65^ can detect multiple markers. These methods analyze HER2 expression levels using mean pixel values extracted from image data. Microarray techniques are capable of handling multiple tissue samples and, with automated analysis, they can evaluate protein expression^59^. Proximity assays^50,60^ have been used to look at HER2 levels and dimerization. The conjugation of quantum dots to anti-HER2 antibodies has provided a novel approach for specifically targeting and visualizing HER2-positive cells^63,64^. Although these methods are intended to improve predictions of patient response to therapy, they typically rely on formalin fixed tissue samples. Because formalin fixation itself requires one day, the approaches are still time intensive. In addition, they provide limited information on the nanoscale organization of HER2 and the presence of the extracellular domain. This type of information may be important for predicting patient response to trastuzumab.

To obtain more comprehensive information without delaying test results, new approaches should be both fast and quantitative. Here, we have used qSMLM on touch prep samples taken from breast cancer patients. Using fluorescently labeled trastuzumab, we directly detected the amount of HER2 present on patient tumor cells, which allowed us to determine aspects of HER2 nano-organization. The scheme of our touch prep-qSMLM approach is shown in Fig. 3. After surgical excision, specimens were examined by a pathologist and tumor areas were touched to a glass coverslip. The transferred cells were fixed and stained with trastuzumab-AF647. The samples were then imaged and analyzed. The overall timeframe for this process is extremely short. In only three hours, a set of coverslips is prepared to encompass multiple monolayer regions of a single patient tumor. Two to three hours are then needed to acquire images. Finally, approximately one hour is needed to quantify both HER2 density and organization. Thus, touch prep-qSMLM can be performed within one workday.

Using touch prep-qSMLM, we detected the amount of HER2 in patient tissues (Table 1). The average detected HER2 densities determined by qSMLM ranged from 15 to 57 molecules/μm^2^. Importantly, average detected HER2 densities from 6 patients had a significant positive correlation with FISH copy numbers (Fig. 4B). The fraction of ROIs above the HER2 density threshold (corresponding to copy number of six for single-probe HER2 FISH) was also investigated. Only P4 had fewer than ~50% regions above the threshold. This demonstrates the utility of our approach for examining HER2 status. To the best of our knowledge, this is the first time qSMLM has been used as a diagnostic tool on fresh patient tissues. In seven patient samples, distinct distributions of HER2 were observed (Table 1, Supplementary Fig. 11). HER2-positive patients had 2.4 to 4.7 HER2 receptors per cluster and the HER2-negative patient showed no evidence of HER2 clustering. Interestingly, for HER2-positive patients, we observed a correlation between HER2 copy numbers and the average number of detected HER2 receptors per cluster (Fig. 4D). While the sample size is small and the confidence interval is wide, future work will include larger patient cohorts to provide information that may help identify important factors governing HER2 status and trastuzumab response.

Several factors make the touch prep-qSMLM an excellent new tool. Within one day, we can determine the density of HER2 that contains the trastuzumab-binding domain. Additionally, clustering parameters can be quantified: HER2 cluster radius, the number of HER2 receptors per cluster, and the fraction of clustered HER2. Since our touch prep method largely does not disrupt tumor cells, we can easily image large areas of intact cell membranes. In contrast, IHC and other methods that employ thin sections of paraffin-embedded tissue mainly provide an orthogonal view of cell membranes^29,45^. This makes global analysis of HER2 expression challenging. Moreover, touch prep-qSMLM can rapidly characterize multiple tumor regions. This allows us to more thoroughly assess tumor heterogeneity. Since breast tumor heterogeneity can drive treatment responsiveness^66^, robust identification of HER2 features with qSMLM may ultimately complement current clinical techniques.

In summary, qSMLM can quantify the density of trastuzumab-bound HER2 in cultured cells and patient tissues. In these proof of concept experiments, touch prep-qSMLM proves to be a useful tool for investigating HER2 status in patients. The molecular details on density and organization of the extracellular HER2 domain could be therapeutically relevant. This line of experiments may ultimately lead to personalized treatments for HER2-positive patients^67^ and help clarify trastuzumab resistance^68^. Over the long term, touch prep-qSMLM may reduce misdiagnoses, shorten intervention timelines, and improve patient outcomes. Our methodology can be easily extended to other HER2-positive cancers^69^ and biomarkers. Thus, touch prep-qSMLM could become an important tool for personalized medicine.

## Methods

### Coverslip cleaning

Coverslips (25-mm diameter, #1.5; Warner Instruments, Hamden, CT) were cleaned as described before^30^. Clean coverslips were stored in sterile 35-mm tissue culture dishes for touch preparation or cell culture.

### Cell culture

BT-474, SK-BR-3, and MDA-MB-468 cells (American Type Culture Collection, Manassas, VA) were cultured in phenol red-free DMEM supplemented with 10% fetal bovine serum, 1 mM sodium pyruvate, 100 U/ml penicillin, 100 U/ml streptomycin, and 2 mM L-alanyl-L-glutamine. For imaging experiments, cells were seeded on coverslips coated with fibronectin-like engineered protein (25 μg/ml in phosphate-buffered saline [PBS], pH 7.4; Sigma-Aldrich, St. Louis, MO).

### Antibodies and fluorescent dye conjugation

Clinical grade trastuzumab was used (Genetech). Primary anti-cytokeratin 7 (monoclonal; abcam), anti-GATA3 (polyclonal; ThermoFisher), and secondary goat anti-rabbit (polyclonal; abcam) antibodies were used for detection of epithelial cells. Trastuzumab and secondary anti-rabbit antibodies were labeled with Alexa Fluor 647 and 405 (Life technologies), respectively. Dyes contained an *N*-hydroxysuccinidimidyl ester (NHS) group for protein conjugation. A solution containing a 4–6 x excess of dye dissolved in dimethyl sulfoxide was mixed with a solution of 1 mg/ml of antibody in PBS, pH 7.4, with 0.02 M NaHCO_3_. The resulting solution was allowed to react for 30 min at room temperature. The reaction was quenched with 1.5 M hydroxylamine (pH 8.5) for 10 min. Unconjugated dye was removed by passing the solution through a size exclusion chromatography column (Bio-Rad, Hercules, CA). Prior to labeling cells or tissues, labeled antibody was passed through a 300-kDa concentrator to remove any potential aggregates. The concentrations of labeled antibodies were measured by a NanoDrop 1000 (Thermo) and calculated with respect to the dye correction factor. The degree of labeling was calculated with the NanoDrop for each batch of trastuzumab labeled with AF647. NHS labeling can result in a combinatorial distribution of dyes on antibody lysine residues^70^. Moreover, an increased degree of labeling leads to decreased affinity for trastuzumab^71^. Thus, we utilized pH conditions for the coupling reaction to promote preferential labeling of terminal amines and to minimize the labeling of lysine side chains. Approximately one dye per antibody was obtained in all cases for trastuzumab labeled with AF647 (degree of labeling determined by the NanoDrop). To minimize effects related to labeling heterogeneity, we defined the average number of detected localizations for trastuzumab-AF647 (Supplementary Fig. 2). As in all experiments that use NHS coupling of dyes to antibodies, very small amounts of antibodies may not be efficiently labeled.

### Immunocytochemistry

After a 2 day seed on coverslips, cells were fixed in PBS with 4% (w/v) paraformaldehyde and 0.2% (w/v) glutaraldehyde (Electron Microscopy Sciences) for 30 min at room temperature. Fixative was quenched with 25 mM glycine in PBS for 10 min and cells were washed three times with PBS. Fixed cells were incubated in blocking buffer (BB; 5% bovine serum albumin [BSA] and 0.1% Tween-20 in PBS) for 20 min. Subsequently, cells were incubated for 1 h with 50 or 10 nM trastuzumab-AF647. After five PBS washes, cells were postfixed for 10 min with 4% (w/v) paraformaldehyde and 0.2% (w/v) glutaraldehyde and inactivated with 25 mM glycine for 10 min at room temperature. For control experiments, six-fold molar excess of HER2 protein (R&D systems, 1129-ER-050) was preincubated with trastuzumab-AF647 for 30 min at room temperature and all subsequent steps were performed as described above. Coverslips were placed in Attofluor cell chambers (Life Technologies) and imaged immediately after preparation in with buffer containing: 50 mM Tris (pH 8.0), 10 mM NaCl, 10% glucose, mercaptoethylamine (100 mM), and glucose oxidase and catalase (GLOX; 10% v/v) as previously described^72^.

### Tissue touch preparation and immunohistochemistry

Cleaned coverslips were coated with poly-L-lysine solution (Sigma-Aldrich, St. Louis, MO), washed, and dried. Tumor tissue was scraped and collected onto the blade of a scalpel, and brought into contact with poly-L-lysine coated coverslips. Tissue on the coverslips was allowed to incubate at room temperature for 5 min to facilitate adhesion. Tissue sample were then fixed in PBS with 4% (w/v) paraformaldehyde and 0.2% (w/v) glutaraldehyde for 30 min at room temperature. This was followed by quenching with 25 mM glycine in PBS for 10 min and washing three times with PBS. Fixed tissue samples were incubated in BB for 20 min. After a wash, tissues were incubated for 1 h with 50 nM trastuzumab-AF647 and primary antibody, anti-GATA3 or anti-cytokeratin 7. Subsequently, tissues were extensively washed with PBS and incubated with 2 μg/ml labeled secondary antibody (with Alexa Fluor 405) for 45 min. After additional PBS washing, tissues were postfixed for 10 min with 4% (w/v) paraformaldehyde and 0.2% (w/v) glutaraldehyde and inactivated with 25 mM glycine for 10 min at room temperature. For control experiments, six-fold molar excess of HER2 protein was preincubated with trastuzumab for 30 min at room temperature and all subsequent steps were performed as just described. Coverslips were imaged immediately after preparation in the same manner as described for immunocytochemistry. Tissue samples were collected under Institutional Review Board (IRB) number 16424 and informed consent was obtained from all subjects.

### Optical setup and imaging acquisition

Measurements were performed on a 3D N-STORM super-resolution microscope (Nikon, Melville, NY) configured for TIRF. The N-STORM system (Nikon Instruments) consists of a fully automatic Ti-E inverted microscope with piezo stage on a vibration isolation table with a 100×/1.49 numerical aperture TIRF objective (Apo), an N-STORM lens and λ/4 lens, and a Quad cube C-NSTORM (97355; Chroma, Bellows Falls, VT) with filters for 405-, 488-, 561-, and 640-nm light. The microscope is equipped with a Perfect Focus Motor to maintain imaging on the desired focal plane, an MLC-MBP-ND laser launch with 405-, 488-, 561-, and 647-nm lasers (Agilent, Santa Clara, CA), and an electron-multiplying charge-coupled device camera (iXon DU897-ultra; Andor Technology, South Windsor, CT). Data were acquired using NIS Elements 4.3 software with automatic drift correction. Laser powers used to activate and/or image dyes were 120 mW (~1.5 kW/cm^2^) and 5–10 mW (~0.06–0.13 kW/cm^2^) measured out of the optical fiber for 647- and 405-nm, respectively. We acquired 20,000–40,000 frames for cells and 20,000 frames for tissues using an exposure time of 10 ms.

All samples were imaged in TIRF mode, and trans-light was used to observe selected regions. For tissue imaging, epithelial cells were identified by scanning at low-power with the 405-nm laser. We adjusted the TIRF angle and focus to image tissue regions close to the coverslip. 647-nm signal coincided with GATA3 or cytokeratin 7 signal. 405-nm channel imaging of Alexa Fluor 405 was performed after the dSTORM acquisition.

### Image analysis

Data processing was performed using NIS-Elements 4.3 software. The minimum number of photons was set to 700. Identification settings for individual localizations were as follows: 200 nm minimum peak width, 400 nm maximum peak width, 300 nm initial fit width, 1.3 maximum axial ratio, and 1 pixel maximum displacement. The peak height threshold was set at 5,000 for AF647. A density filter was applied (70 nm radius and 50 counts) to remove artificial clusters^73^ in tissues (Supplementary Fig. 12). For consistency, the same density filter was applied to cells, although few, if any, artifacts were observed.

After data processing, localization density and protein auto-correlation functions were computed in MATLAB (Natick, MA). Briefly, binary images of cells or tissues were prepared using localization xy-coordinate centers obtained from NIS-elements. Localizations corresponding to noise were first removed from these images via thresholding. Those localizations with a precision (σ) outside the 98^th^ percentile of values were discarded. (Histograms in Supplementary Figs 3 and 9 reflect the results of this thresholding step). Binary images were appropriately generated from binned localizations. Following some optimization, a binned pixel size of 1.6 nm ensured that no more than ~1% of detected localizations were lost to this process. The binary images then included binned pixels with localizations assigned to a value of 1 and all other pixels with no localizations were set to 0. Next, multiple square regions of interest (ROIs) of 20 μm^2^ were placed across areas of positive signal corresponding to cells or tissue (confirmed through brightfield images) and densities were calculated. The total number of localizations from within these regions was divided by a constant value α to obtain detected densities in terms of the number of molecules. Here, α represents the average number of discrete appearances (localizations) of trastuzumab-AF647. This value is obtained using MDA-MB-468 cells as described in Supplementary Fig. 2. Multiple ROIs were collected and averaged for many individual cells for all patients and cell lines (Table 1 and Supplementary Table 1). Density distributions for all ROIs are shown in Supplementary Figs 4 and 10.

To evaluate the robustness of our density data, we used a strategy similar to that previously reported for other biophysical approaches^74^. We randomly split all ROIs for individual patients or cell line types into two groups and calculated the p-values for densities between the two groups (p-value_split_). This was accomplished by first randomizing all of the density values for a given patient using the *rand* function in MATLAB. Subsequently, the Student’s T-Test was performed in Excel using a one-tailed distribution with a heteroscedastic two-sample unequal variance type. In all cases, no significant difference in density was observed between two groups (p-value_split_ ≥ 0.1). The results for patients are provided in Table 1 and the results for cell lines are provided in Supplementary Table 1.

Auto-correlation functions were computed using fast Fourier transforms using the previously published algorithm^36,37^ (equation [1]) to obtain the number of proteins per cluster and cluster radius. This is illustrated in Fig. 1A, middle panel (results are summarized in Table 1 and Supplementary Table 1). A k-means-like clustering algorithm^38,39^ was subsequently used to quantify the fraction of clustered receptors (more than two HER2 receptors per cluster). For each ROI with detected HER2 clusters, this algorithm takes into account the average localization precision and cluster radius from PC analysis to define the fraction of HER2 receptors in clusters. Local clusters are identified across the ROI by grouping localizations into molecules via this PC cluster radius and maximum fluorophore dark time^72^. Molecules were counted as part of a cluster if they meet these spatiotemporal requirements. Otherwise, molecules were labeled as unclustered. MATLAB code calculating the extent of clustering is provided (Supplementary Software: ClusterOccupancy) and an example ROI image is shown in Fig. 1A, right panel. Results are summarized in Table 1 and Supplementary Table 1.

### Monte Carlo Simulations

Synthetic localization data was generated to validate HER2 information extracted from PC analysis and a k-means-like clustering algorithm on BT-474 and SK-BR-3 cell data (50 nM trastuzumab-AF647 staining). Input parameters and other variables, and their use within MATLAB code, were as follows:

#### Number of appearances per trastuzumab-AF647 (α)

This variable was set to an average of 3, the same value obtained from experimental data (Supplementary Fig. 2), and allowed to vary according to a Poisson distribution (MATLAB *poissrnd* function). This distribution was subsequently used to help provide the random number of localizations associated with an individual molecule.

#### Localization precision (σ)

This variable was set to an average of 12 nm, the same value obtained from experimental data (Supplementary Fig. 3 and Supplementary Table 1), and allowed to vary according to a Normal distribution (MATLAB *normrnd* function). This distribution was further used to help provide random error in the positions of localizations associated with an individual molecule.

#### Number of photons per localization

This was set to an average of 2000 as obtained from experimental data. Photons for an individual localization in a given frame were drawn from a Poisson distribution with a minimum threshold of 700.

#### Number of molecules per cluster

This was set to an average of either 3 or 2 (for BT-474 or SK-BR-3 cells, respectively) and allowed to vary from 1.5 to 4.5 as this range represented the majority of values observed from PC analysis (Supplementary Fig. 7A).

#### Cluster radius

This was set to an average of either 20 or 24 nm (BT-474 or SK-BR-3 cells, respectively) and allowed to vary from 18 to 32 nm as this range represented the majority of values observed from PC analysis (Supplementary Fig. 7B).

#### Density (localizations/μm^2^)

This was set equal to the experimentally observed average number of localizations within a 20 μm^2^ ROI (360 or 230 locs/μm^2^ for BT-474 or SK-BR-3 cells, respectively) and allowed to fluctuate using the MATLAB *rand* function. Variation in the total number of localizations was derived from the data presented in Supplementary Fig. 4.

### Statistical considerations

Data were summarized by reporting mean values, SEM, and coefficients of variation (CV). Pearson’s correlation coefficient (r) was used to evaluate the correlation between detected HER2 density (from qSMLM) and copy number (from FISH). Additionally, Pearson’s correlation coefficient was used to evaluate the correlation between the average number of HER2 receptors per cluster and copy number. A 95% confidence interval for the correlation coefficient was also presented. The least square regression line was fitted to the data to help visualize the correlation.

## Electronic supplementary material

Supplementary Information
