# Supplementary material for: Single molecule localization microscopy coupled with touch preparation for the quantification of trastuzumab-bound HER2
Source: Sci Rep. 2018 Oct 11;8:15154. doi: 10.1038/s41598-018-33225-0 (PMC6181918; doi:10.1038/s41598-018-33225-0)
Supplement: Supplementary file 1 — Supplementary Information [file 41598_2018_33225_MOESM1_ESM.pdf]

Supplementary Information

Single molecule localization microscopy coupled with touch preparation for the quantification of trastuzumab-bound HER2

Steven J. Tobin, Devin L. Wakefield, Veronica Jones, Xueli Liu, Daniel Schmolze, Tijana Jovanović-Talisman

Supplementary Figure 1

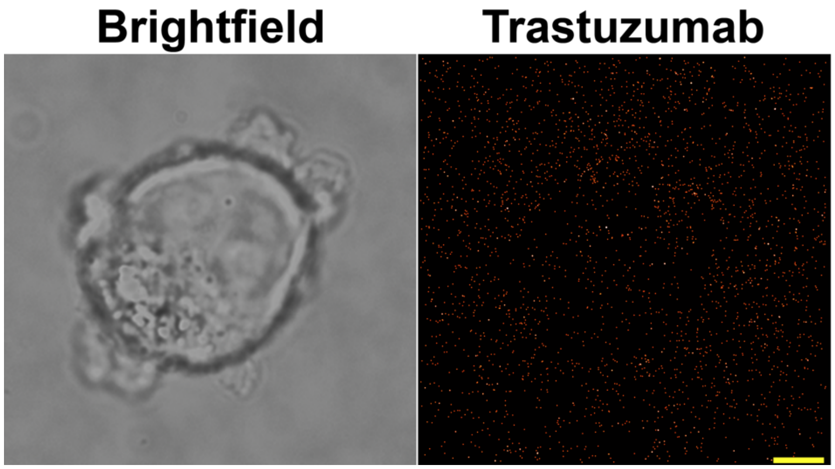

Supp. Fig. 1. Trastuzumab-AF647 is specific for HER2 in breast cancer cell lines. HER2 protein was preincubated with 50 nM trastuzumab-AF647 and applied to cells. Negligible SMLM signal was observed on all coverslips. A representative image of a BT-474 cell incubated with HER2 and trastuzumab-AF647 complex is shown; brightfield image (left) and direct stochastic optical reconstruction microscopy (dSTORM) image (right).

Supplementary Figure 2

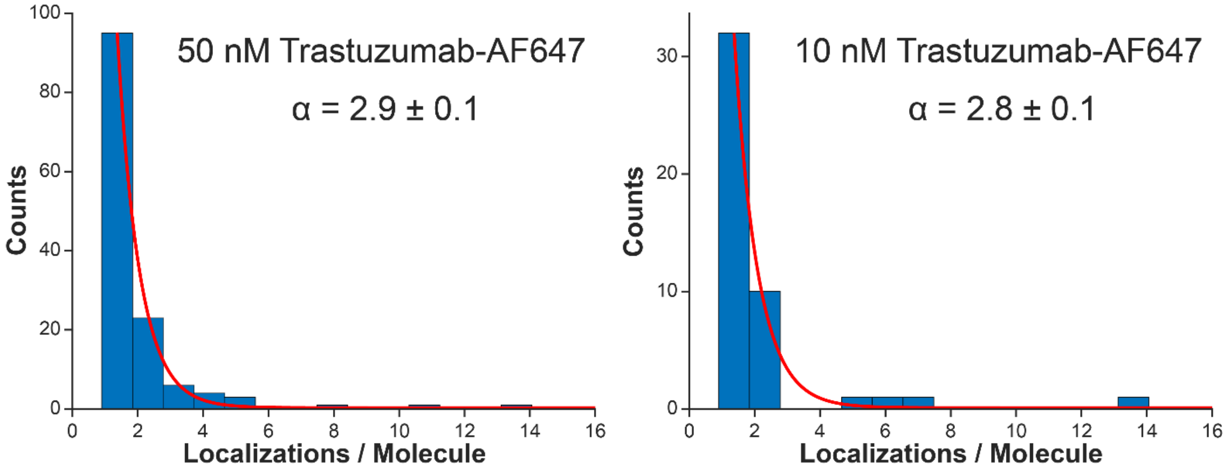

Supp. Fig. 2. Average number of localizations per molecule for trastuzumab-AF647. Sparse trastuzumab-AF647 staining in MDA-MB-468 cells was used to obtain the average number of appearances (α). Detected localizations within a region of interest (64 μm^2) were first grouped (assigned to a single molecule) using spatial and temporal thresholds: a resolution-limited distance and a temporal window equal to the maximum fluorophore dark time[1]. Localizations were assigned to a new molecule if both criteria were not met. Histograms for the distribution of these grouped localizations is provided here for trastuzumab-AF647 staining at 50 nM (21 cells) and 10 nM (9 cells). A single exponential fit (red line) is applied to extract the value for the average number of localizations (appearances) for a single trastuzumab-AF647 Ab.

Supplementary Figure 3

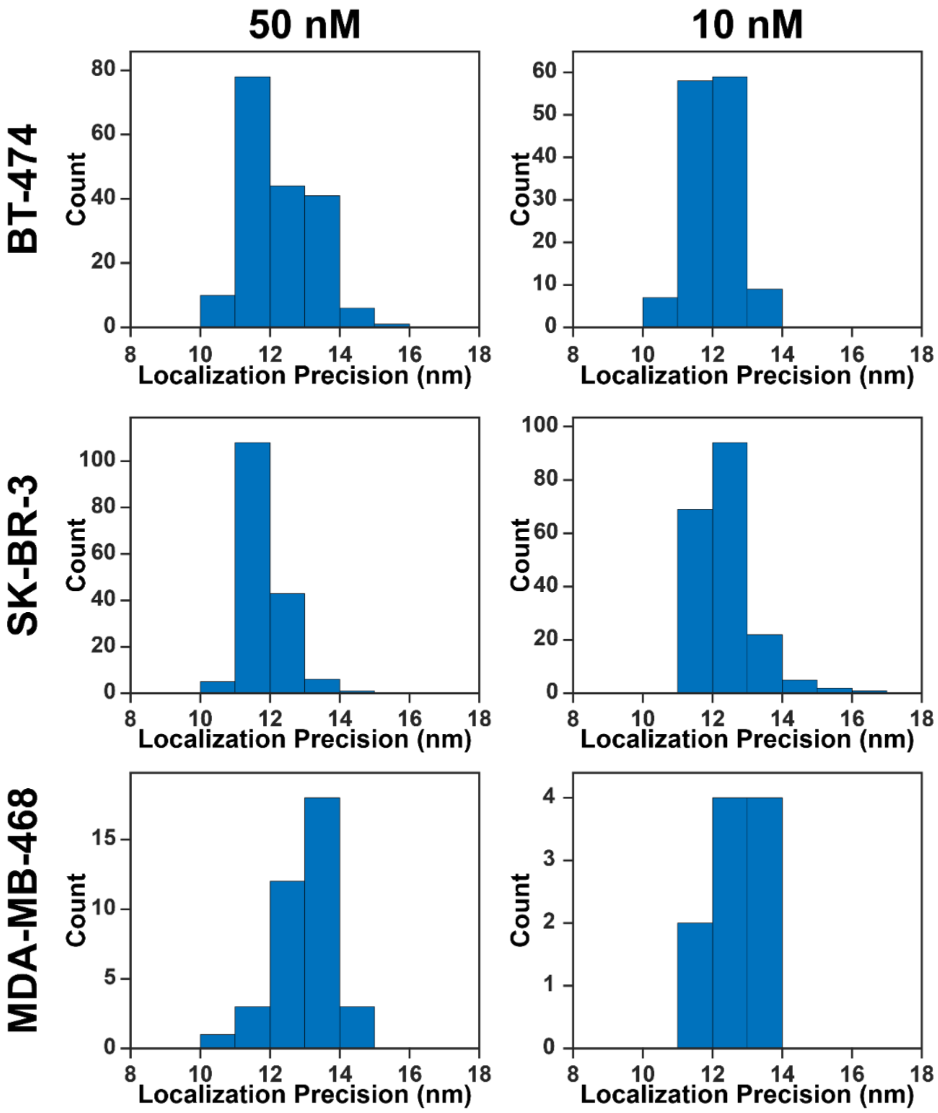

Supp. Fig. 3. Localization precision (σ) distributions from breast cancer cell lines. Cells were stained with either 50 or 10 nM trastuzumab-AF647. σ is provided for all ROIs used for data analysis.

Supplementary Figure 4

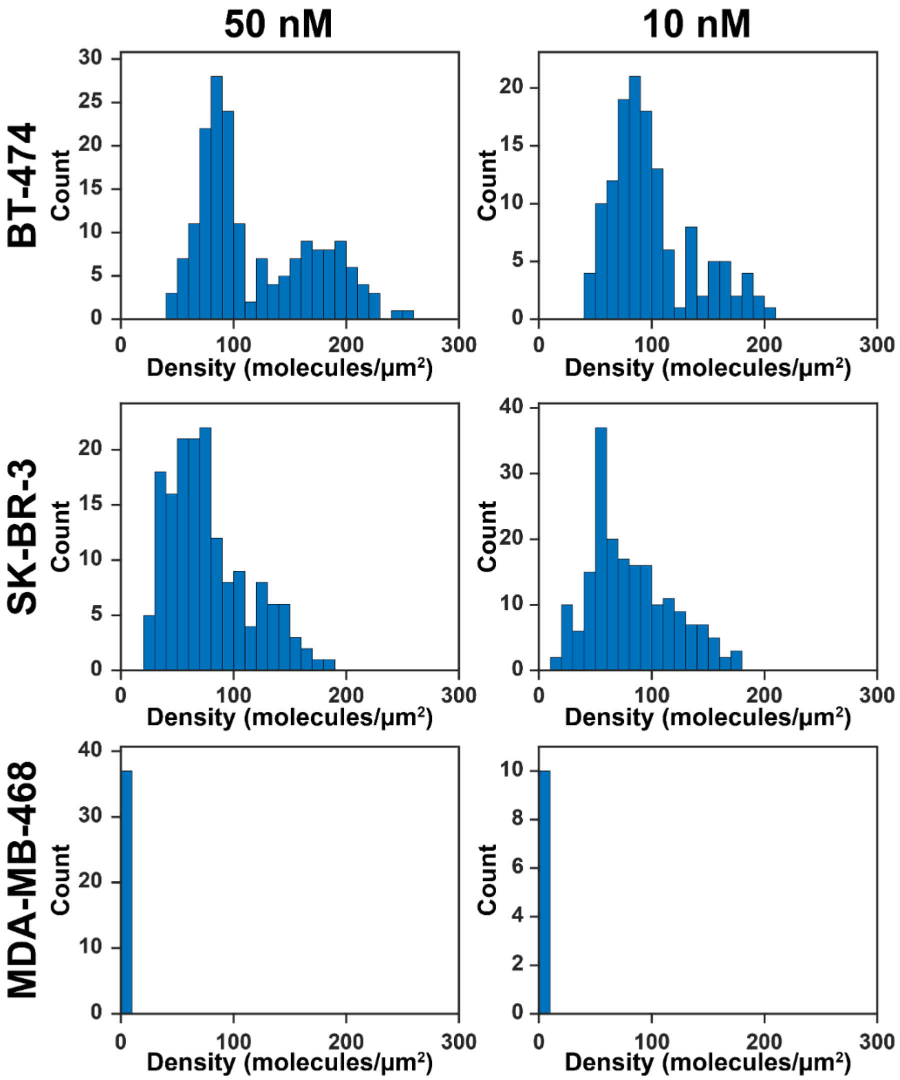

Supp. Fig. 4. Density distributions from breast cancer cell lines. Cells were stained with either 50 or 10 nM trastuzumab-AF647. Density is provided for all reported ROIs.

Supplementary Figure 5

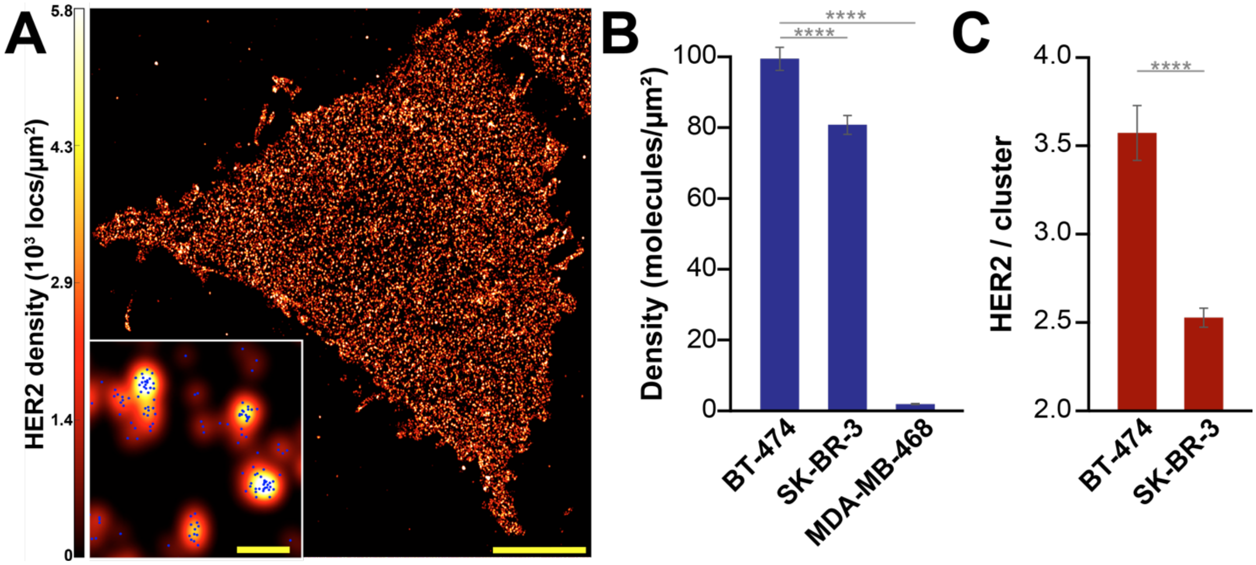

Supp. Fig. 5. HER2 molecular features in breast cancer cell lines stained with 10 nM trastuzumab-AF647. BT-474, SK-BR-3, and MDA-MB-468 cells were fixed, stained with 10 nM trastuzumab-AF647, and imaged with dSTORM. **A.** A BT-474 cell with individual localizations processed from NIS-Elements (blue dots) and rendered by a Gaussian blur. The intensity of the Gaussian blur is proportional to the localization density. Scale bar: 5 µm. A zoomed-in region is provided as an inset (scale bar: 100 nm). **B.** Average detected HER2 density for BT-474 cells (18 cells,133 ROIs), SK-BR-3 cells (20 cells, 193 ROIs), and MDA-MB-468 cells (5 cells 10 ROIs); ****$p < 0.0001$. **C.** Average number of HER2 receptors per cluster for BT-474 and SK-BR-3 cells calculated using pair-correlation (PC) analysis. Clustered regions were used for analysis (57 ROI for BT-474 and 114 ROI for SK-BR-3); ****$p < 0.0001$. All error bars represent SEM.

Supplementary Figure 6

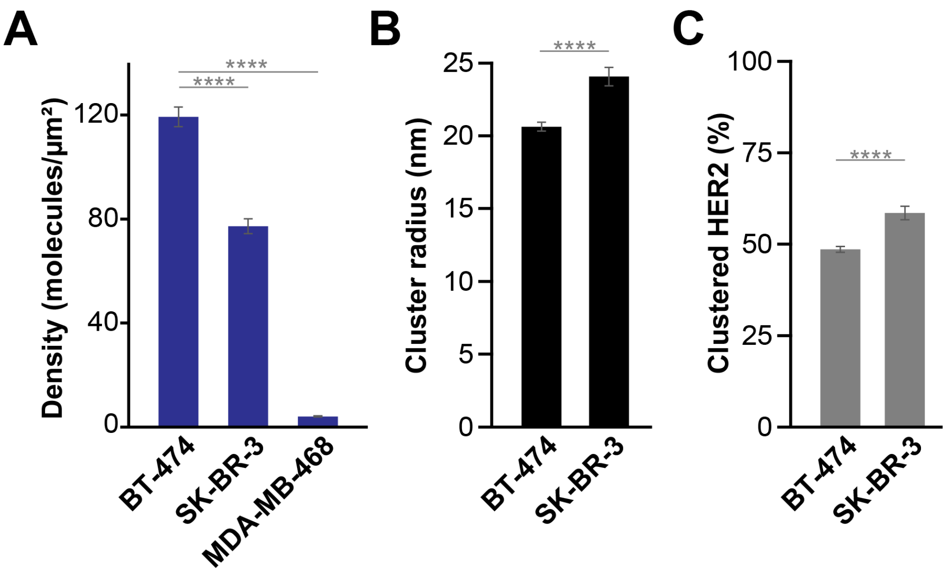

Supp. Fig 6. Additional HER2 molecular features in breast cancer cell lines stained with 50 nM trastuzumab-AF647. A. Average detected HER2 density (as presented in Fig. 1C). p-value$_{split}$ values: 0.5 for BT-474 cells, 0.3 for SK-BR-3 cells, 0.2 for MDA-MB-468 cells. **B.** Average HER2 cluster radius. **C.** The average fraction of HER2 molecules residing in clusters with more than two receptors. For **B** and **C**, clustered regions were used for analysis (53 ROI for BT-474 and 31 ROI for SK-BR-3); ****p < 0.0001. All error bars represent SEM.

Supplementary Figure 7

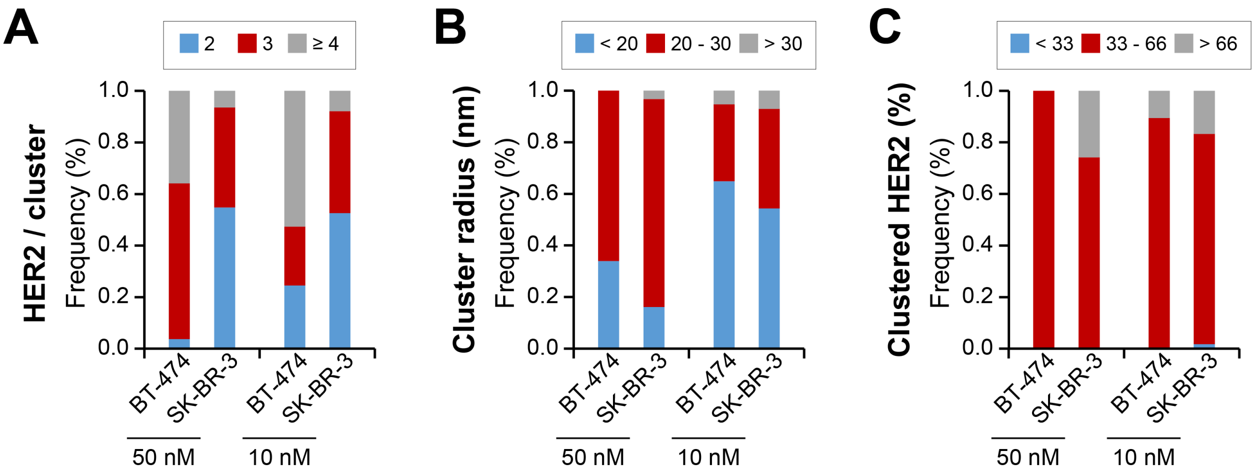

Supp. Fig 7. Distribution of clustering parameters from breast cancer cell lines stained with either 50 nM or 10 nM trastuzumab-AF647. A. Number of HER2 receptors per cluster**. B.** Cluster radius**. C.** Fraction of HER2 molecules residing in clusters with more than two receptors.

Supplementary Figure 8

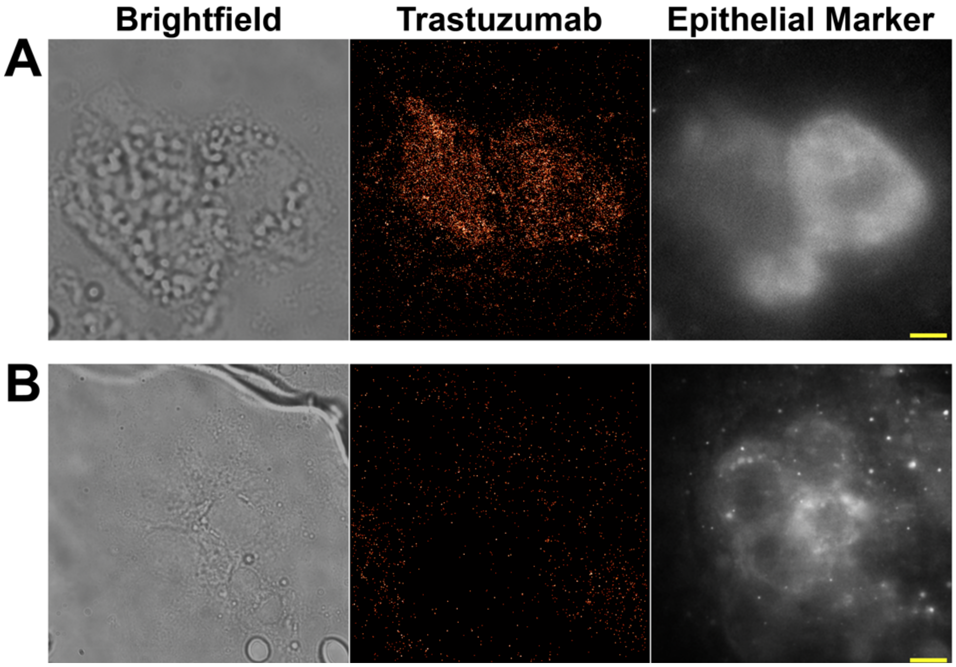

Supp. Fig. 8. Trastuzumab-AF647 is specific for HER2 in breast cancer tissues. A. Brightfield, dSTORM (detecting trastuzumab-AF647), and TIRF (detecting AF405 labeled epithelial marker) image of a tissue sample region from P3. **B.** Coverslip with tissue sample from P5 was incubated with HER2 and trastuzumab-AF647 complex; brightfield image (left), dSTORM image (middle), and TIRF image (right). Negligible SMLM signal was observed on all investigated coverslips. Scale bars: 5 μm.

Supplementary Figure 9

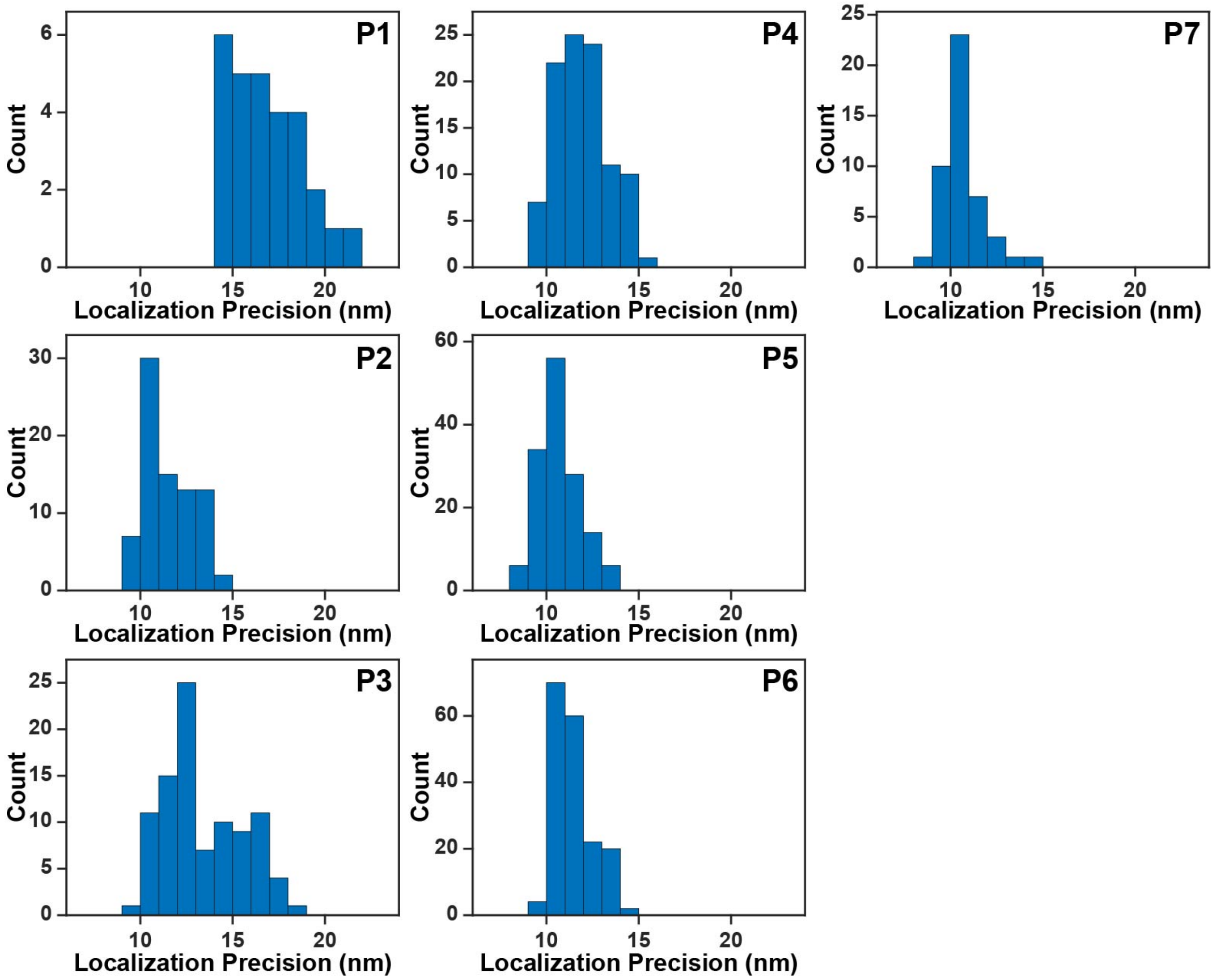

Supp. Fig. 9. Localization precision (σ) distributions from breast cancer tissues.
σ is provided for all ROIs used for data analysis.

Supplementary Figure 10

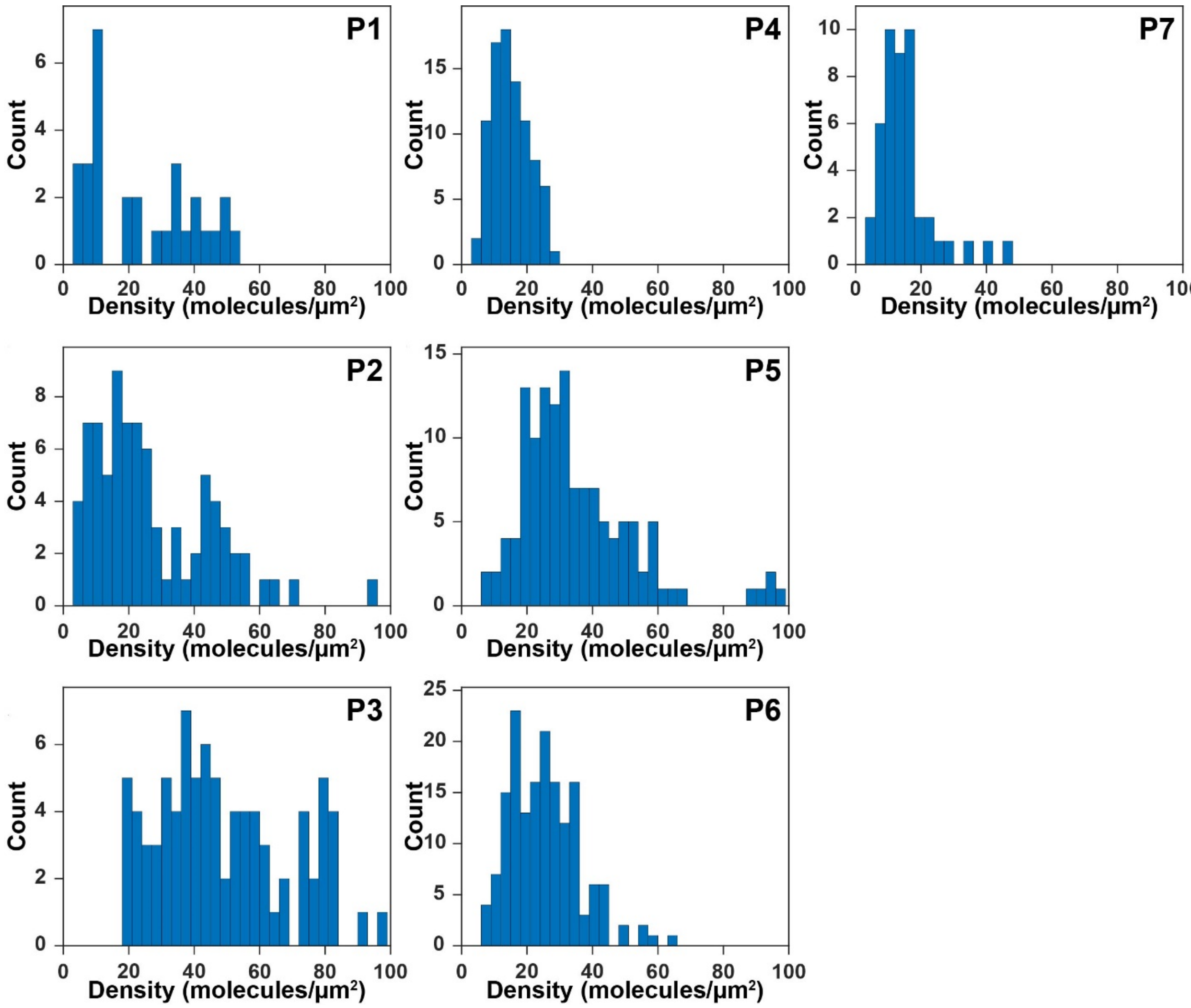

Supp. Fig. 10. Density distributions from breast cancer tissues. Density is provided for all reported ROIs.

Supplementary Figure 11

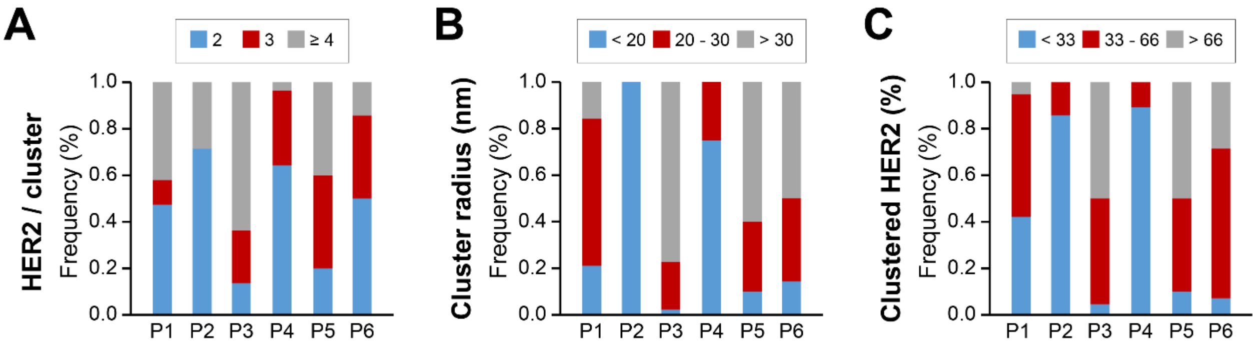

Supp. Fig 11. Distribution of clustering parameters from breast cancer tissues. A. Number of HER2 receptors per cluster**. B.** Cluster radius**. C.** Fraction of HER2 molecules residing in clusters with more than two receptors.

Supplementary Figure 12

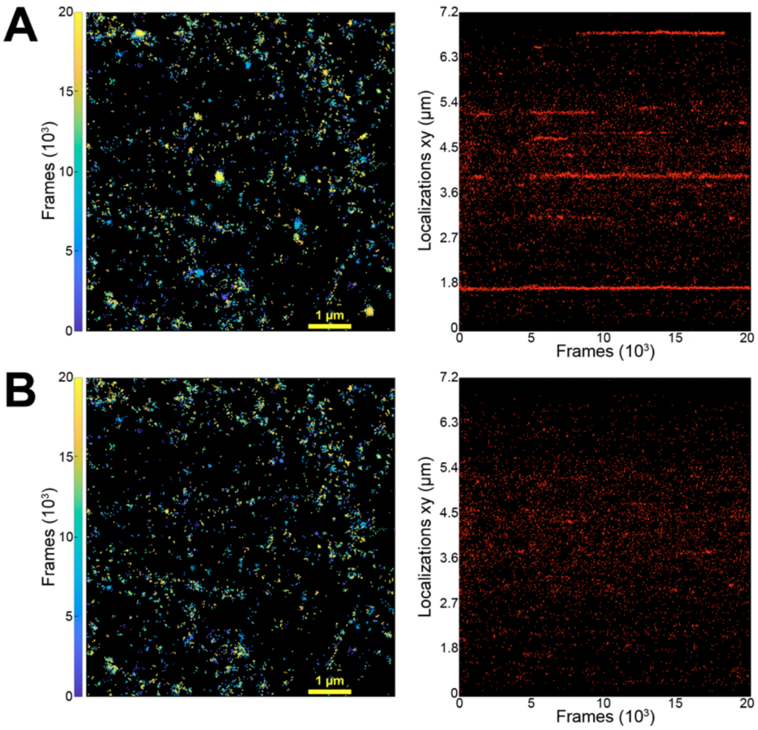

Supp. Fig. 12. Density filtering of artificial clusters. A. An ROI from a patient tissue sample is shown with localizations color-coded according to their time of appearance (left). Localization coordinates plotted as a function of their time of appearance reveal the presence of artificial clusters[2] (right). **B.** The application of a density filter (radius of 70 nm with a minimum of 50 localizations) shows that these artificial clusters are removed.

Supplementary Table 1

	50 nM Trastuzumab			10 nM Trastuzumab		
	**BT-474**	**SK-BR-3**	**MDA-MB-468**	**BT-474**	**SK-BR-3**	**MDA-MB-468**
**Density (molecules/μm^2) ± SEM**	119 ± 4	77 ± 3	4 ± 0.2	99 ± 3	81 ± 3	2 ± 0.2
CV (%)	42	46	29	38	46	29
**HER2 / cluster ± SEM**	3.3 ± 0.1	2.5 ± 0.1	monomer	3.6 ± 0.2	2.5 ± 0.1	monomer
CV (%)	14	23	N/A	33	23	N/A
**Cluster radius (nm) ± SEM**	21 ± 1	24 ± 1	N/A	20 ± 1	21 ± 1	N/A
CV (%)	11	15	N/A	21	24	N/A
**Clustered HER2 (%) ± SEM**	49 ± 1	59 ± 2	0	54 ± 1	54 ± 1	0
CV (%)	12	18	N/A	15	21	N/A
**Localization precision (nm) ± SEM**	12.3 ± 0.1	11.8 ± 0.1	13.0 ± 0.2	12.0 ± 0.1	12.4 ± 0.1	12.6 ± 0.3
CV (%)	8	5	7	5	7	7
**Coverslips**	4	3	4	3	4	3
**ROIs**	180	163	37	133	193	10
**p-value$_{split}$**	0.5	0.3	0.2	0.4	0.3	0.1

Supp. Table 1. Breast cancer cell line data summary. Average values for detected density, HER2 receptors per cluster, cluster radius, fraction of clustered HER2, and localization precision are provided with SEM and CV.

References:

1 Dempsey, G. T., Vaughan, J. C., Chen, K. H., Bates, M. & Zhuang, X. W. Evaluation of fluorophores for optimal performance in localization-based super-resolution imaging. *Nat Methods* **8**, 1027-1036, doi:10.1038/nmeth.1768 (2011).

2 Annibale, P., Vanni, S., Scarselli, M., Rothlisberger, U. & Radenovic, A. Identification of clustering artifacts in photoactivated localization microscopy. *Nat Methods* **8**, 527-528, doi:10.1038/nmeth.1627 (2011).

Supplementary Software: ClusterOccupancy

MATLAB code for computing the fraction of clustered and unclustered molecules within a single region of interest.

```
function [pfm, pcm, pfo, pco] = ClusterOccupancy(ROI,ROIArea,MPC,CR,GR,FG,showFig)
% Calculate cluster occupancy of ROI (fraction of clustered and unclustered molecules)
% Apply DP-Means algorithm to define clusters

% Input:
% ROI = ROI data [X Y Sigma Frames Photons]
% ROIArea = ROI area (ensure same units as X Y)
% MPC = minimum number of molecules to count as part of a cluster, default 3
% CR = cluster radius (PC analysis correlation length), default estimated from X Y
% GR = group radius (gaussian fit to localization precision data), default
prctile(Sigma,98)
% FG = frame group gap, default AF647 at 150s -> 150s*(100f/s exposure time) = 15000
frames
% showFig = show/hide figure with ROI data and cluster occupancy

% Output:
% pfm = '%unclustered' molecules
% pcm = '%clustered' molecules
% pfo = '%unclustered' area occupancy
% pdo = '%clustered' area occupancy

%-------------------------------------------------------------------------------------
--------------
%% Organize ROI data and check/prepare variables
X = ROI(:,1); Y = ROI(:,2);
Sigma = ROI(:,3);
Frames = ROI(:,4);
Photons = ROI(:,5);

if ~exist('MPC', 'var') || ~MPC, MPC = 3; end
if ~exist('GR', 'var') || ~GR, GR = prctile(Sigma,98); end
if ~exist('FG', 'var') || ~FG, FG = 15000; end
if ~exist('showFig', 'var') || isempty(showFig), showFig = true; end
if ~exist('CR', 'var') || ~CR
    [m,n] = size([X Y]); %estimate CR from coordinate data
    CR = 0.75*((prod(max([X Y])-min([X Y]))*gamma(0.5*n+1))/(m*sqrt(pi.^n))).^(1/n);
end

%% Group localizations (peakclustering) and extract clsuters (DP_means)
cM = peakclustering([X Y Sigma Sigma Frames],GR,FG,Photons);

%Identify clusters via spatial data and DP-Means algorithm
[Cxy, cID] = DP_means(cM,CR,mean(cM));

%% Plot ROI data
if showFig
    scnsize = get(0,'ScreenSize');
    figure('OuterPosition',[1 40 scnsize(3)*0.52 scnsize(4)*0.95],'Name','ROI
Occupancy Map','Color','w')
    subplot('Position', [0, 0, 1, 0.95])
    plot(X,Y,'b.','MarkerSize',5)
    axis([min(X)*0.997 max(X)*1.003 min(Y)*0.997 max(Y)*1.003])
    set(gca,{'Color','XTick','YTick'},{'k','',''})
    for i = 1:size(cM(:,1),1) %show grouped coordinates
        radius = GR/3; cir = [cM(i,1)-radius*1.5,cM(i,2)-
radius*1.5,radius*3,radius*3];
        rectangle('Position',cir,'Curvature',[1,1],'LineWidth',0.5,'EdgeColor','r')
```

```
    end
end

%% Calculate fraction of unclustered and clustered molecules
Nunclustered = 0; Nfm = 0; unclusteredA = 0; %number of unclustered molecules
'clusters', number of unclustered molecules, unclustered area
Nclusters = 0; Ncm = 0; ClusterA = 0; %number of clusters with CR, number of clustered
molecules, cluster area
for i = 1:size(Cxy(:,1),1)
    distance_to_mean = pdist2(cM(cID==i,:),Cxy(i,:));
    cr(i) = max(GR,max(distance_to_mean)); %cluster radius
    cir = [Cxy(i,1)-cr(i),Cxy(i,2)-cr(i),2*cr(i),2*cr(i)];
    if size(cM(cID==i,:),1) < MPC % unclustered molecules
        if showFig,
rectangle('Position',cir,'Curvature',[1,1],'LineWidth',1.5,'EdgeColor','g')
        end
        Nunclustered = Nunclustered + 1;  Nfm = Nfm + size(cM(cID==i,:),1);
unclusteredA = unclusteredA + pi*cr(i)^2;
    elseif size(cM(cID==i,:),1) >= MPC %clustered molecules
        if showFig,
rectangle('Position',cir,'Curvature',[1,1],'LineWidth',1.5,'EdgeColor',[0.5 0 1])
        end
        Nclusters = Nclusters + 1;  Ncm = Ncm + size(cM(cID==i,:),1);  ClusterA =
ClusterA + pi*cr(i)^2;
    end
end
pfm = (Nfm/(Ncm + Nfm))*100;  %'% unclustered'
pfo = (unclusteredA/ROIArea)*100;    %'% unclustered' area
pcm = (Ncm/(Ncm + Nfm))*100;  %'%clustered'
pco = (ClusterA/ROIArea)*100; %'%clustered' area

if showFig %figure title with details, add scalebar
    title({['Molecules:  {\color{green}' num2str(pfm,4) '% (' num2str(Nfm) ')
Unclustered}  {\color[rgb]{0.6,0,1}' ...
        num2str(pcm,4) '% (' num2str(Ncm) ') Clustered}'];...
        ['Occupancy:  {\color{green}' num2str(pfo,3) '% Unclustered}
{\color[rgb]{0.6,0,1}' ...
        num2str(pco,3) '%
Clustered}']},'Color','w','BackgroundColor','k','EdgeColor','k','FontSize',12)
end

disp(['Localizations: ' num2str(length(X))])
disp(['ROI area: ' num2str(ROIArea)])
disp(['Unclustered molecules: ' num2str(Nfm) ' => ' num2str(pfm,4) '%'])
disp(['Unclustered occupancy: ' num2str(pfo,4) '%'])
disp(['Number of clusters: ' num2str(Nclusters)])
disp(['Clustered molecules: ' num2str(Ncm) ' => ' num2str(pcm,4) '%'])
disp(['Cluster occupancy: ' num2str(pco,4) '%'])
disp(' ')

%------------------------------------------------------------------------------------
--------------
%% Supporting functions: 'peakclustering' and 'DP_means'

function [cK, iK, sK] = peakclustering(D,RG,FG,P)
% Assign initial clusters for DP-Means

% Input:
% D = [X, Y, SigmaX, SigmaY, Frames]
% RG = radius gap
% FG = frame gap
% P = photons
```

```
% Output:
% cK = cluster centers
% iK = cluster indices
% sK = cluster sigmas

%-------------------------------------------------------------------------------------
--------------
K = 0; %number of clusters
D = sortrows(D,5); %ensure frame numbers increase
iK = zeros(size(D,1),1); %cluster indicator
xK = zeros(K,1);  yK = zeros(K,1); %cluster xy-centers
sxK = zeros(K,1); syK = zeros(K,1); %cluster xy-sigma
ia = false(K,1); %active clusters
fgap = zeros(K,1); %frame gap counter

fnum = D(1,5); %frame number
for i = 1:size(D,1) %total number of localizations
        if D(i,5) > fnum %check for frame advance
           fgap = fgap+D(i,5) - fnum; %increase frame gap counter
           fnum = D(i,5);
        end

        ia(fgap >= FG) = false; %check for terminated clusters
        xi = D(i,1);   yi = D(i,2);
        sxi = D(i,3); syi = D(i,4);
        if ~isempty(ia)
             dist = sqrt((xK(ia)-xi).^2 + (yK(ia)-yi).^2)/RG; %euclidean norm, good
match
            [dmin,imin] = min(dist);
        else
            dmin = inf;
        end
        if  isempty(ia) || (sum(ia)==1 && fgap(K)==0) %clusters terminated/new frame
            dmin = inf;
        end
        if  dmin < 1 %assign to existing cluster
            nK = (1:K); nK = nK(ia); %active cluster indices
            jK = nK(imin); %assigned cluster
            iK(i) = jK;
            fgap(jK,1) = 0; %reset frame gap counter of assigned cluster
            %update cluster means and sigmas
            itemp = iK == iK(i); % points in updated cluster

            xK(jK) = P(itemp)'*D(itemp,1)/sum(P(itemp)); %photon weighted average of
x-values
            yK(jK) = P(itemp)'*D(itemp,2)/sum(P(itemp)); %photon weighted average of
y-values
            sxK(jK) = std(D(itemp,1));
            syK(jK) = std(D(itemp,2));

        else %create new cluster
            K = K+1;  iK(i) = K;
            xK(K,1) = xi;    yK(K,1) = yi;
            sxK(K,1) = sxi;  syK(K,1) = syi;
            ia(K,1) = true; %make new cluster active
            fgap(K,1) = 0;  %initialize frame gap counter for new cluster to 0
        end
end
cK = [xK yK];
sK = [sxK syK];

function [cXY, cID] = DP_means(cM,lambda,cMm)
```

```
% Adapted from Kulis & Jordan 2012, "Revisiting k-means: New Algorithms via Bayesian
Nonparametrics"
% Algorithm 1 in paper

% Input:
% cM = grouped localization centers from peakclustering
% lambda = cluster radius
% cMm = cluster seed coordinates

% Output:
% cXY = cluster coordinates
% cID = cluster indices

%-------------------------------------------------------------------------------------
--------------
K = 1; %initial number of clusters
n = size(cM,1); %number of localizations
iter = 0; %run algorithm at least once
c_old = zeros(n,1); criterion_met = 0; %stop criteria, met when c == c_old
lambda_criterion = 1; %Assign data to nearest cluster: satisfied when there are no
points lambda away from the nearest cluster

h = waitbar(0,'DP-Means: Analyzing cluster occupancy ...');
while criterion_met == 0 || lambda_criterion == 1
    iter = iter + 1;
    distance_to_mean = pdist2(cM,cMm); %Euclidean distance
    %distance_to_mean = pdist2(Mc,Mcm,'chebychev'); %Chebychev distance (aka, infinity
norm) (max coordinate difference)

    %Assign clusters
    [min_value_vec, index] = min(distance_to_mean,[],2);  cID(1:n,1) = index;

    criterion_met = 0;
    if all(eq(cID,c_old)) %ensure k-means has converged before adding a new cluster
        criterion_met = 1; %k-means converged, now add cluster if needed
        [~,ind] = max(min_value_vec); %index of outlier
        lambda_criterion = any(min_value_vec > lambda);
        if lambda_criterion > 0  %create new cluster (at least lambda away from
nearest cluster)
            K = K+1; cID(ind,1) = K;
            cMm = [cMm; cM(ind,1:2)]; %create new cluster for outlier
        end
    end

    %calculate means (https://statinfer.wordpress.com/2011/12/12/efficient-matlab-ii-
kmeans-clustering-algorithm/)
    E = sparse(1:n,cID,1,n,K,n);                %transform label into indicator matrix
    cMm = (E*spdiags(1./sum(E,1)',0,K,K))'*cM;  %compute cluster matrix
    c_old = cID;

    waitbar(K/n,h)
end
close(h)
```
